# Accident vasculaire cérébral ischémique large chez un sujet jeune révélateur d’une endocardite infectieuse: à propos d’un cas

**DOI:** 10.11604/pamj.2016.25.31.10347

**Published:** 2016-09-27

**Authors:** Fanomezantsoa Noella Ravelosaona, Julien Razafimahefa, Rahamefy Odilon Randrianasolo, Solofonirina Rakotoarimanana, Djacoba Alain Tehindrazanarivelo

**Affiliations:** 1 Service de Neurologie, CHUJR Befelatanana, Antananarivo; 2 Unité de Soins Intensifs en Cardiologie, CHUJR Befelatanana, Antananarivo

**Keywords:** AVC ischémique, antibiothérapie, endocardite infectieuse, sujet jeune, Ischemic stroke, antibiotic therapy, infective endocarditis, about young

## Abstract

L’Accident vasculaire cérébral ischémique large est le plus souvent du à un embole d’origine cardiaque ou à partir d’une plaque d’athérome. Chez le sujet jeune, l’une des principales causes d’AVC ischémique surtout large est la cardiopathie emboligène dont l’endocardite infectieuse. Or, l’endocardite infectieuse est une contre indication de l’anticoagulation efficace indiquée lors d’une cardiopathie emboligène compliquée d’un AVC ischémique. L’une des complications cérébrales de l’endocardite infectieuse est l’AVC ischémique mais souvent de localisation multiple. Nous rapportons l’observation d’un homme de 44 ans qui a présenté une hémiplégie gauche massive d’apparition brutale dans un contexte apyrétique, associé à un souffle mitral systolique et qui est devenu fébrile à J5 d’hospitalisation sans autre foyer infectieux évident. Le scanner cérébral a montré un accident vasculaire ischémique large du territoire de l’artère sylvienne totale droite et l’échocardiographie doppler à distance de l’accident a montré une endocardite infectieuse de la petite valve mitrale. Il a été traité par une biantibiothérapie pendant 4 semaines sans anticoagulation et l’évolution était marquée par la disparition des végétations sur la valve mitrale et par les séquelles motrices de l’hémicorps gauche. Notre problème en pratique était la survenue de la fièvre non concomitante ni précédant le déficit entrainant une errance dans le diagnostic vers un AVC ischémique d’origine cardio-embolique. Ce tableau souligne l’intérêt de faire une échocardiographie doppler dans tout AVC ischémique large surtout superficiel avant tout traitement anticoagulant.

## Abstract (en)

Broad ischemic stroke is mainly due to a cardiac embolus or to an atheromatous plaque. In young subjects, one of the main causes of ischemic stroke (broad ischemic stroke in particolar) is embolic heart disease including infective endocarditis. Infective endocarditis is a contraindication against the anticoagulant therapy (which is indicated for the treatment of embolic heart disease complicated by ischemic stroke). One neurologic complications of infective endocarditis is ischemic stroke which often occurs in multiple sites. We here report the case of a 44-year old man with afebrile acute onset of severe left hemiplegia associated with a sistolic mitral murmur, who had fever in hospital on day 5 with no other obvious source of infection present. Brain CT scan showed full broad ischaemic stroke of the right middle cerebral artery territory and doppler ultrasound, performed after stroke onset, showed infective endocarditis affecting the small mitral valve. He was treated with 4 weeks of antibiotic therapy without anticoagulant therapy ; evolution was marked by the disappearance of mitral valve vegetations and by movement sequelae involving the left side of the body. In practical terms, our problem was the onset of the fever which didn’t accompany or pre-exist patient’s deficit, leading us to the misdiagnosis of ischemic stroke of cardioembolic origin. This case study underlines the importance of doppler ultrasound, in the diagnosis of all broad ischemic strokes, especially superficial, before starting anticoagulant therapy.

## Introduction

Les complications neurologiques au cours d’une endocardite infectieuse sont fréquentes avec une incidence à 50% si pas de prise en charge précoce [1]. L’Accident vasculaire cérébral ischémique (AVC) large par embolie d’une végétation constitue l’une des principales complications et peut ainsi caché une endocardite infectieuse sous jacente. Le traitement est basé sur une antibiothérapie et l’anticoagulation efficace est contre indiquée devant cette situation [1]. Nous rapportons un cas atypique d’endocardite infectieuse révélé par un AVC ischémique large chez un sujet jeune et nous discutons des problèmes posés lors de sa prise en charge en pratique.

## Patient et observation

**Figure 1:**
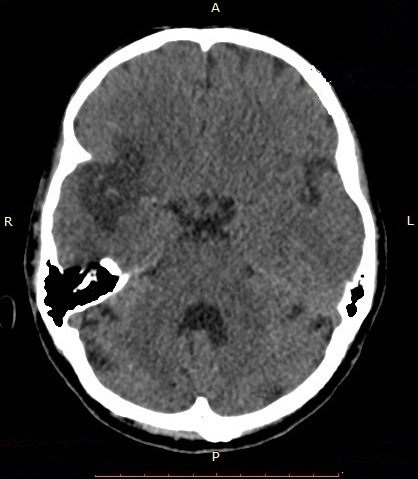
Coupe TDM cérébrale axiale sans injection d’un patient de 44 ans hospitalisé pour hémiplégie gauche montrant la visibilité spontanée de l’artère sylvienne droite

**Figure 2:**
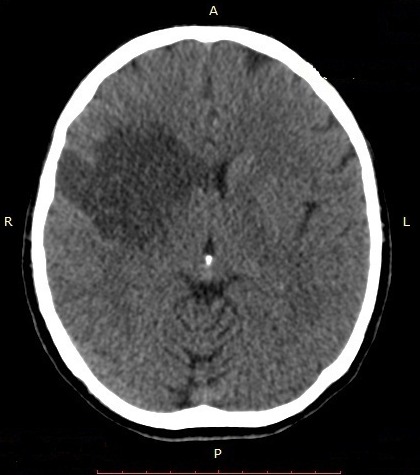
Coupe TDM cérébrale axiale sans injection d’un patient de 44 ans hospitalisé pour hémiplégie gauche montrant une hypodensité large touchant la substance blanche et substance grise du territoire de l’artère sylvienne totale droite

**Figure 3:**
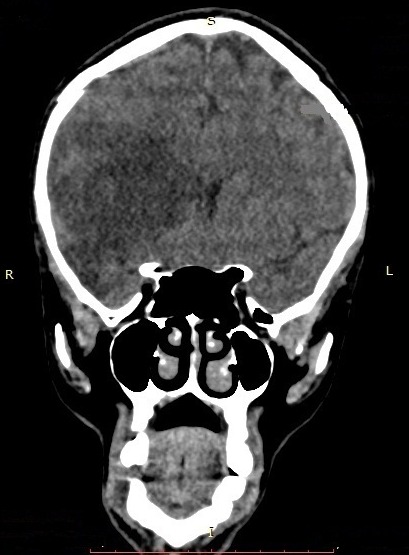
Coupe TDM cérébrale coronale sans injection d’un patient de 44 ans hospitalisé pour hémiplégie gauche montrant une hypodensité large touchant la substance blanche et substance grise du territoire de l’artère sylvienne totale droite

**Figure 4:**
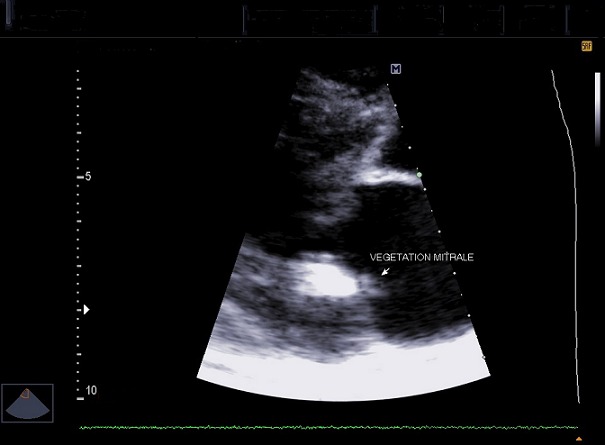
Échocardiographie doppler cardiaque réalisée à J14 d’hospitalisation montrant de végétation au niveau petite valve mitrale

**Figure 5:**
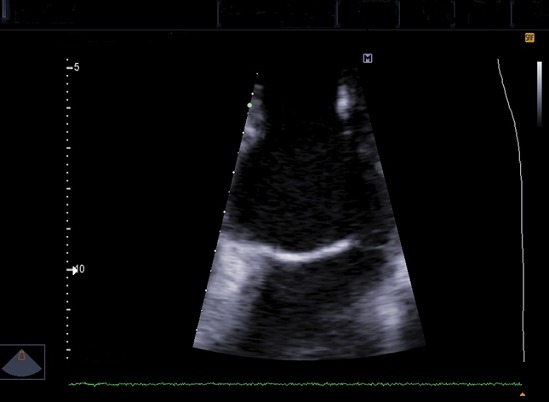
Échocardiographie doppler cardiaque de contrôle réalisée après 4 semaines de traitement montrant la disparition de végétation au niveau petite valve mitrale

Il s’agit d’un homme de 44 ans qui a été hospitalisé au service de Neurologie du CHU/JRB Antananarivo pour une hémiplégie gauche. Pour l’histoire de la maladie il a présenté brutalement une impotence fonctionnelle de l’hémicorps gauche massive sans céphalée ni trouble de la conscience ni agitation et il n’y avait pas de notion de fièvre. Dans son antécédent, angine à répétition dans l’enfance et mal traitée, pas de cardiopathie connue. L’examen clinique à l’admission a montré un bon état général, une apyrexie. Un score NIHSS à 15 et échelle Rankin à 4, une conscience normale, un déficit sensitivo-moteur type pyramidal de l’hémicorps gauche, sans trouble de la déglutition ni sphinctérien. Ailleurs, présence d’un souffle systolique d’intensité 3/6 au niveau foyer mitral, pas de signe d’insuffisance cardiaque, un mauvais état bucco-dentaire, pas d’atteinte cutanée ni articulaire. A J5 d’hospitalisation apparition d’une fièvre à 39-40°C sans autres foyers infectieux évidents. Il n’y avait pas de syndrome infectieux biologique avec CRP à 12 mg/l, hémoculture 2 séries et sérologie HIV étaient négatives, fond d’œil normal. Le scanner cérébral avec injection à J7 hospitalisation a montré une visibilité spontanée de l’artère sylvienne droite (Figure 1) et une hypodensité large touchant substance blanche et grise du territoire de l’artère sylvienne totale droite (Figure 2, Figure 3). Ce qui nous a amené à demander un échocardiographie doppler qui n’a été réalisée qu’à J14 qui a objectivé la présence de petite valve mitrale remaniée calcifiée avec des végétations de 12X 16 mm et 6, 37X 9,07mm (Figure 4) et FEVG conservée à 82,20%. L’hypothèse diagnostique était une endocardite infectieuse de la petite valve mitrale compliquée d’un AVC ischémique large du territoire de l’artère sylvienne totale droite. Le traitement institué était une biantibiothérapie: association d’un Beta-lactamine et un aminoside et également une kinésithérapie motrice quotidienne. L’évolution était marquée par une apyrexie à 24h du traitement, bonne observance et tolérance thérapeutique. L’échocardiographie doppler de contrôle après 4 semaines de traitement a montré la disparition totale des végétations (Figure 5) mais cliniquement persistance du déficit moteur.

## Discussion

Cette observation nous rappelle que l’AVC ischémique large est une complication spécifique d’une endocardite infectieuse et constitue même un contexte révélateur chez le sujet jeune. Dans plusieurs études européennes, l’AVC ischémique constitue 20 à 60% des complications neurologiques de l’endocardite infectieuse mais la forme large reste rare mais non méconnue surtout au niveau du territoire de l’artère cérébrale moyenne [2]. Souvent le tableau clinique est celui d’un déficit moteur associé à une fièvre et un souffle cardiaque d’emblée et s’ensuit la découverte d’un AVC au scanner cérébral et d’une endocardite infectieuse sous jacente à l’échocardiographie doppler en même temps. Mais pour notre cas la découverte de l’endocardite infectieuse était plus tardive vue que qu’il n’y avait pas de fièvre qu’en cours d’hospitalisation. Mais le point faible réside sur le fait que l’hémoculture était négative, en effet l’identification du germe constitue un facteur pronostic important [3]. Néanmoins, le traitement médical sans anticoagulation permettait une disparition complète des végétations et sans passage à la chirurgie.

## Conclusion

Devant un tableau d’impotence fonctionnelle d’apparition brutale associé à un souffle cardiaque même sans fièvre concomitante ni syndrome infectieux une endocardite infectieuse sous jacente est possible et faire avant tout une échocardiographie doppler. En effet, le traitement antibiothérapie sera spécifique sans nécessité d’un traitement anticoagulant ni chirurgical.
